# Sex Differences in the Quality of Diabetes Care in the Netherlands (ZODIAC-45)

**DOI:** 10.1371/journal.pone.0145907

**Published:** 2015-12-29

**Authors:** Steven H. Hendriks, Kornelis J. J. van Hateren, Klaas H. Groenier, Sebastiaan T. Houweling, Angela H. E. M. Maas, Nanne Kleefstra, Henk J. G. Bilo

**Affiliations:** 1 Diabetes Centre, Isala, Zwolle, the Netherlands; 2 Department of General Practice, University of Groningen and University Medical Center Groningen, Groningen, the Netherlands; 3 Langerhans Medical Research Group, Zwolle, the Netherlands; 4 Department of Cardiology, Radboud University Medical Center, Nijmegen, the Netherlands; 5 Department of Internal Medicine, University of Groningen and University Medical Center Groningen, Groningen, the Netherlands; 6 Department of Internal Medicine, Isala, Zwolle, the Netherlands; University of Glasgow, UNITED KINGDOM

## Abstract

**Objective:**

Our aim was to investigate whether trends in quality of diabetes care differ between sexes in the Netherlands from 1998 till 2013.

**Research Design and Methods:**

In this prospective observational cohort study quality of care was measured using process and outcome measures in patients with type 2 diabetes in primary care. Trend and absolute differences between sexes were investigated for patients <75 years. Subgroup analyses were performed in patients ≥75 years. 10-year mortality risk was assessed with the Globorisk risk equation in patients without cardiovascular diseases <75 years.

**Results:**

The number of patients increased from 2,644 in 1998 to 62,230 in 2013. In 1998, 51% of the men and 60% of the women <75 years had an HbA1c >53 mmol/mol; this decreased to approximately 29% in both sexes in 2013. Patients having a systolic blood pressure >140 mmHg decreased from 70% to 42%, and from 80% to 40% in men and women <75 years, respectively. In patients ≥75 years it decreased from 72% to 50% in men and 85% to 56% in women. Obesity increased in both sexes, whereas smoking in men and women declined in patients <75 years (men: 34% to 22%; women: 22% to 18%). The number of patients with a mortality risk >20% over 10 years decreased from 15% to 3% in men and from 18% to 3% in women.

**Conclusions:**

Quality of diabetes care has improved considerably in the period 1998–2013 in both sexes. Possibly relevant trend differences between sexes were observed for HbA1c, systolic blood pressure, BMI and smoking. The predicted mortality risk decreased over time in both sexes. Except for BMI in both age groups and systolic blood pressure in patients ≥75 years, no evident poorer risk factor control in women compared to men was found at the end of the study period.

## Introduction

It is reported in the literature that the risk of cardiovascular mortality is increased about twofold in men and threefold in women with type 2 diabetes (T2D) compared with men and women without T2D [1,2]. A poorer control of cardiovascular risk factors in women with T2D compared to men is considered as a possible explanation for this difference [1]. Some studies indicate that target levels for clinical parameters are less frequently achieved in women [3–7]. In an Italian study for example, the target value for HbA1c (<53 mmol/mol) was achieved in 34% of women compared to 40% of men [3]. The percentage of women who achieved the target value for systolic blood pressure appears to be 2 to 4% lower than in men [3–5]. Some studies indicate sex disparities in treatment intensity as the explaining factor for this difference in risk factor control whereas others describe that this difference could be explained by psychosocial mechanisms, like patient compliance [5,6].

Taken together, it could be that there is a difference in quality of care for men and women with T2D. One way to assess quality of diabetes care is by using quality indicators. Quality of care is hereby captured in process and outcome measures which are based on national and/or international guidelines. Process measures indicate the number of patients in which a physical examination or laboratory test is performed and an outcome measure reflects the actual results of the assessments and interventions. Measuring the same process and outcome measures over time makes it possible to measure changes in diabetes care and to investigate if the changes differ between sexes over time. The existence of possible differences may indicate that there should be more emphasis on sex-specific diabetes care.

A previous study from our study group showed that quality of diabetes care has considerably improved in the period 1998–2008 [8]. Possible sex disparities and whether these disparities have changed over time have not been investigated in our previous study. Investigating sex differences in this study makes it possible to measure trends in possible sex differences, instead of measuring cross-sectional differences only which has been done in most of the previous studies. Therefore, the aim of the current study was to investigate whether trends in quality of diabetes care differed between sexes in the Netherlands from 1998 until 2013 in patients <75 years and in the subgroup of patients >75 years of age.

## Materials and Methods

This study uses, to some extent, the methodology as published before [8].

### Study population

The study population consisted of patients who are included in the Zwolle Outpatient Diabetes project Integrating Available Care (ZODIAC) project. This project started in 1998 as a study to investigate the effects of shared care for patients with T2D who are treated in primary care [9]. Due to positive results, this shared care initiative became the standard care for the Zwolle region in 2002. In 2006, 2009 and 2012, it expanded to other regions which are connected to our diabetes centre for benchmark and research purposes.

Only T2D patients treated in primary care are included in the ZODIAC project. Patients with a very short life expectancy or insufficient cognitive capabilities are excluded from participation. At the start in 1998, 53 general practitioners (GPs) participated in this project, and this number increased to 731 GPs in 2013. All patients who were participating for at least one year in the ZODIAC project between 1998 and 2013 were included in the current study.

### Data collection and outcome measures

Quality measures, based on the guidelines of the Dutch College of General Practitioners and the Dutch Diabetes Federation, are collected in the general practitioners’ (GP) patient information systems, mostly by practice nurses. As part of the shared care initiative, clinical data are uploaded and sent to our Diabetes Centre for benchmarking and research purposes annually. All general practices use the same system to upload their data about the patients with T2D. In the first years, practice nurses had to fill in the information system manually. Nowadays, most of the information is automatically extracted from the patient information systems of the general practices.

In the current study, process and outcome measures were assessed. All process and outcome measures are depicted in Table 1.

**Table 1:** Process and outcome measurements.

Parameter	Process measure	Outcome measure
HbA1c	Percentage of patients measured	Mean HbA1c (mmol/mol)
		Percentage HbA1c >53 mmol/mol
Diabetes treatment	Not applicable	Percentage diet only
		Percentage oral medication only
		Percentage insulin with or without oral medication
Systolic blood pressure	Percentage of patients measured	Mean SBP (mmHg)
		Percentage SBP ≥140 mmHg
Hypertension treatment	Not applicable	Percentage patients using
		antihypertensive drugs
Cholesterol-HDL ratio	Percentage of patients measured	Mean cholesterol-HDL ratio
		Percentage cholesterol-HDL ratio ≥4
Lipid lowering treatment	Not applicable	Percentage of patients using lipid lowering drugs
Renal function	Percentage of patients with ACR measurements	Percentage microalbuminuria: Women: ACR 3.5–35 mg/mmol; Men: ACR 2.5–25 mg/mmol
		Percentage macroalbuminuria: Women: ACR >35 mg/mmol; Men: ACR >25 mg/mmol
Foot examination	Percentage of patient examined. (Tested 5.07 Semmes-Weinstein monofilaments).	Percentage of patients with diminished sensibility. (Defined as two or more errors in a test of three per foot).
Eye examination	Percentage of patients with retinascreening (investigated with a retinal camera).	Percentage of patients with DRP.
BMI	Percentage of patients measured	Mean BMI (kg/m^2^)
		Percentage BMI ≥25 kg/m^2^
		Percentage BMI 25–30 kg/m^2^
		Percentage BMI ≥30 kg/m^2^
Smoking	Percentage of patients asked	Percentage of smokers

Abbreviations: SBP, systolic blood pressure; HDL, high-density lipoprotein; ACR, albumin-creatinine ratio; DRP, diabetic retinopathy; BMI, body mass index.

To estimate the 10-year mortality risk based on a part of the outcome measures, the Globorisk risk equation was used [10]. This risk prediction equation includes sex, age, smoking, blood pressure, total cholesterol and diabetes to predict 10-year cardiovascular mortality risk. Mortality risk was assessed in patients <75 years of age without a history of cardiovascular disease (CVD). Not having CVD was defined as not using thrombocyte aggregation inhibitors and not having a registration for a history of angina pectoris, myocardial infarction, percutaneous transluminal coronary angioplasty, coronary artery bypass grafting, stroke or transient ischemic attack in the GPs’ patient information system. A country-specific risk chart for the Netherlands was not available so therefore the risk chart of Denmark, a country with a more or less comparable population composition and comparable quality of diabetes care compared to the Netherlands, was used [11]. Low risk was defined as a cardiovascular mortality risk below 10%, medium risk as a risk between 10 to 20% and a high risk as a risk above 20%.

### Statistical analyses

Main analyses were performed in men and women <75 years of age. Subgroup analyses were performed in men and women ≥75 years of age. Patients were divided in these two age groups to create a comparable mean age for men and women for the visual analyses and because of the limited evidence for the treatment of T2D and cardiovascular risk management in patients aged older than 75 years. Data were expressed as mean or median with 95% confidence interval (CI) for normally distributed and non-normally distributed data, respectively. Nominal variables are represented as the proportion of patients with 95% CI. The descriptive statistics for each year were cross-sectional and included data of all visits. Cross-sectional outcomes tend to overestimate time trends compared to longitudinal analyses [12]. Therefore, the existence of a linear time trend from 1998 to 2013 was tested using a generalized linear mixed model (PROC GLIMMIX) with a normal distribution for continuous variables and a binomial distribution using the logit link function for binary variables, both adjusted for age and sex. Model fit of the (generalized) linear mixed models was measured using a correlation method which was proposed by Zheng et al. [13]. All trends were visually inspected and the results of quadratic trend analysis were only included when such a trend was likely based on the plot and when there was a substantial increase in model fit. Time, age and sex (if applicable) were modelled as fixed effects in the analyses. Since the estimated linear time trends are based on individual changes over time, data of at least two visits were necessary. Trend and absolute differences between both sexes were investigated visually and by adding sex as an interaction term and confounder to the models. Trends were also investigated in stratified analyses according to sex. Trend and absolute differences between sexes were marked as possible relevant when clear differences were observed visually. All descriptive analyses were performed with SPSS V.20 software and all mixed model analyses with SAS V.9.2 software.

### Ethical approval

The ZODIAC study and the informed consent procedure were approved by the local medical ethics committee of the Isala, Zwolle, the Netherlands. In the first years of ZODIAC, verbal informed consent was obtained from all patients and the consent was documented in the patient’s records. According to Dutch law, written informed consent was not necessary for this type of study in 1998. Nowadays, written informed consent is obtained. All data were analysed anonymously.

## Results

**Fig 1:**
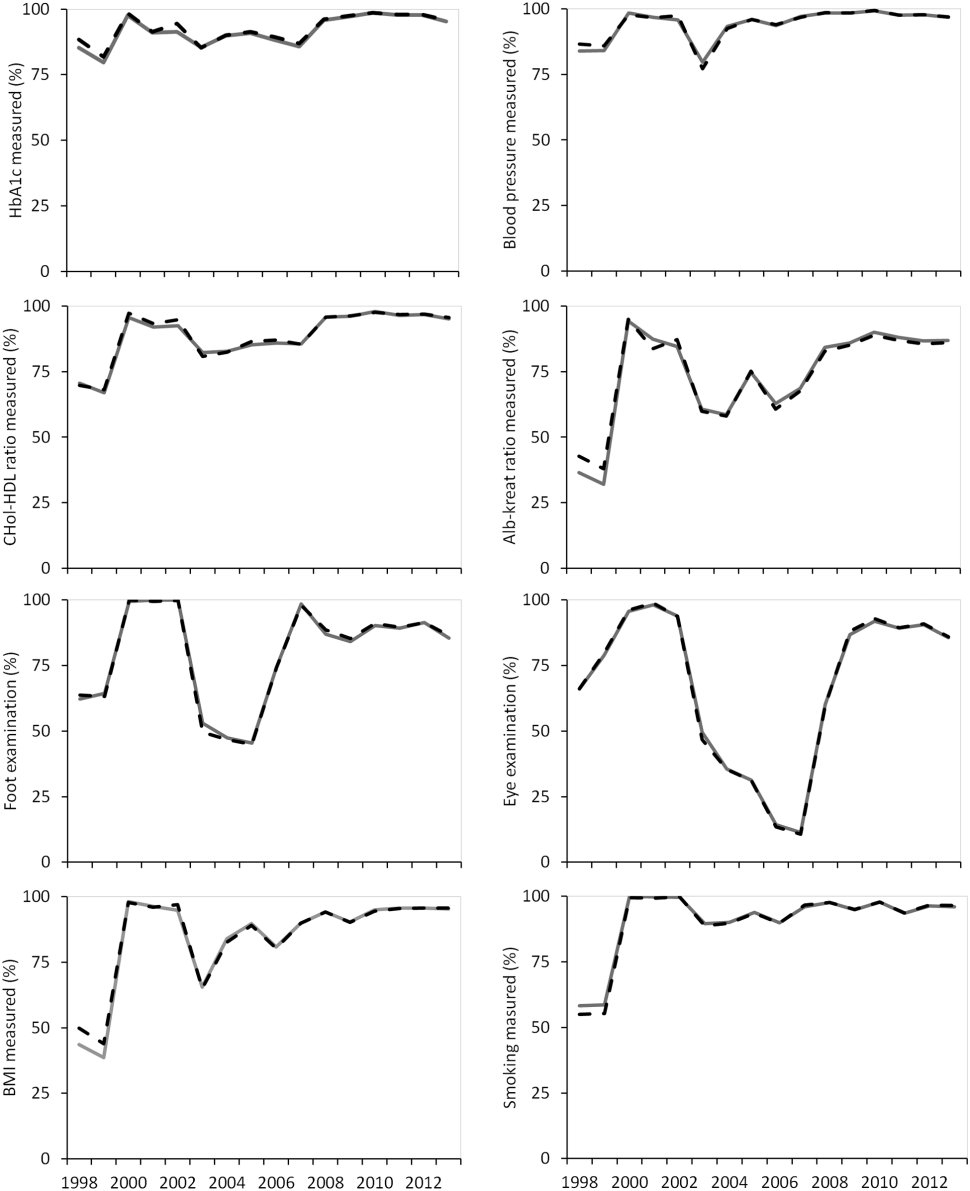
Process measures in patients <75 years stratified according to sex. Men: gray line, Women: black striped line.

**Fig 2:**
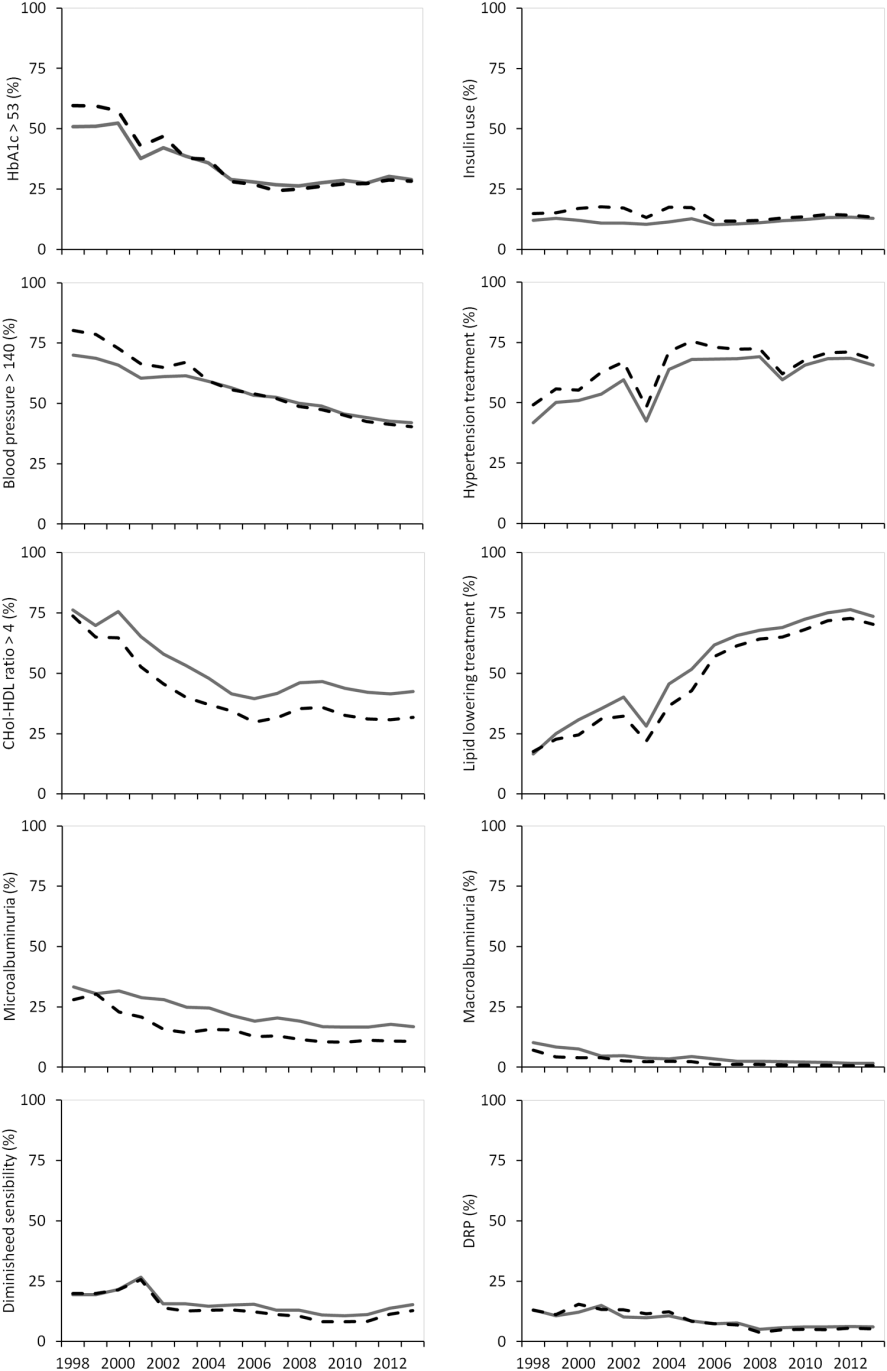
Outcome measures for HbA1c, diabetes treatment, systolic blood pressure, hypertension treatment, cholesterol-HDL ratio, lipid lowering treatment, renal function and foot and eye examination in patients <75 years stratified according to sex. Men: gray line, Women: black striped line.

**Fig 3:**
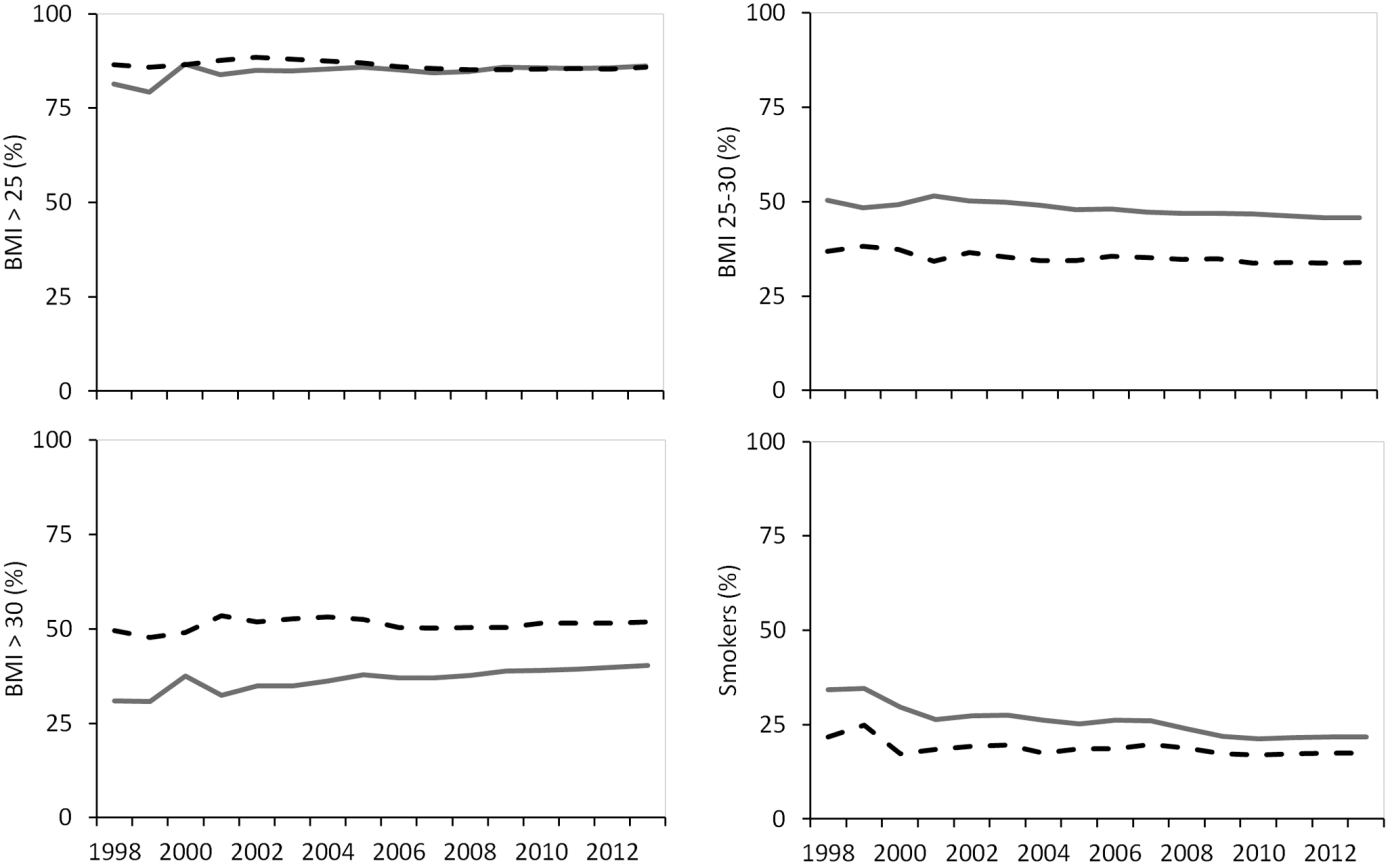
Outcome measures for BMI and smoking in patients <75 years stratified according to sex. Men: gray line, Women: black striped line.

The number of patients with T2D <75 years increased from 1791 in 1998 to 42,641 in 2013. The number of patients ≥75 years increased from 853 to 19,589 in the same period. The proportion of men increased over time to 45.8% in patients <75 years in 2013. Median diabetes duration increased from 4.5 to 6.0 years in patients <75 years. Mean age slightly decreased in women and increased in men during the study period (women <75: 63.3 to 62.4 years, men <75: 61.0 to 62.0 years). The cross-sectional results for all process and outcome measures for patients <75 years are presented in Table 2. Tables stratified for sex are presented in S1 and S2 Tables. The process measures for men and women <75 years are presented in Fig 1 and the outcome measures for men and women <75 years are presented in Figs 2 and 3. The outcome measures for men and women >75 years of age are presented in S1 and S2 Figs.

**Table 2:** Results of the process and outcome measurements for patients under 75 years of age.

Variable	1998	2000	2002	2004	2006	2008	2010	2013	P value for linear trend	P value for int.^#^	P value for gender	R^2^
N	1791	791	1192	4471	12833	19872	30338	42641				
Age (years)	62.1	62.1	61.6	61.9	61.7	61.9	61.8	62.2	<0.001	0.067	<0.001	-
	(61.7–62.6)	(61.5–62.7)	(61.1–62.1)	(61.6–62.1)	(61.6–61.9)	(61.7–62.0)	(61.7–61.9)	(62.1–62.3)				
Sex	50.4	50.6	50.8	48.9	48.3	47.8	46.8	45.8	<0.001	NA	NA	-
(% female)	(48.1–52.7)	(47.1–54.1)	(48.0–53.7)	(47.4–50.3)	(47.4–49.1)	(47.1–48.5)	(46.2–47.3)	(45.3–46.3)				
DM duration	4.5	4.9	4.0	4.4	4.3	4.8	5.1	6.0	<0.001	0.017	<0.001	-
(years)	(4.1–4.7)	(4.4–5.3)	(3.6–4.2)	(4.2–4.5)	(4.2–4.4)	(4.8–4.9)	(5.0–5.2)	(6.0–6.1)				
HbA1c	86.8	98.1	93.0	90.0	88.7	96.0	98.6	95.6	<0.001	0.680	0.078	0.337
process (%)	(85.3–88.4)	(97.2–99.1)	(91.5–94.4)	(89.1–90.8)	(88.2–89.3)	(95.7–96.3)	(98.5–98.8)	(95.4–95.8)				
HbA1c mean	57.5	56.8	53.8	52.0	49.9	49.2	50.2	50.2	<0.001	<0.001	0.021	0.728
(mmol/mol)	(56.8–58.2)	(55.9–57.8)	(53.0–54.6)	(51.7–52.4)	(49.7–50.1)	(49.0–49.3)	(50.0–50.2)	(50.1–50.3)				
HbA1c >53	55.3	54.9	44.6	36.5	27.4	25.7	27.9	28.6	<0.001*	<0.001	<0.001	0.926
mmol/mol (%)	(52.8–57.8)	(51.4–58.4)	(41.7–47.5)	(35.0–38.0)	(26.6–28.3)	(25.0–26.3)	(27.4–28.4)	(28.2–29.1)				
DM treatment	13.8	13.0	19.6	18.3	21.4	20.5	19.6	17.2	<0.001*	0.873	0.649	0.577
Diet only (%)	(12.2–15.4)	(10.7–15.4)	(17.4–21.9)	(17.2–19.5)	(20.6–22.1)	(19.9–21.0)	(19.1–20.0)	(16.9–17.6)				
OBLD only (%)	66.0	67.1	61.5	63.3	63.9	64.0	64.0	62.8	0.586	0.529	<0.001	0.244
	(63.8–68.2)	(63.9–70.4)	(58.7–64.3)	(61.9–64.7)	(63.1–64.3)	(63.3–64.7)	(63.4–64.5)	(62.3–63.2)				
Insulin (%)	13.5	14.5	14.1	14.4	11.0	11.5	13.0	13.1	0.519	<0.001	<0.001	0.072
	(11.9–15.1)	(12.1–17.0)	(12.1–16.1)	(13.4–15.4)	(10.4–11.5)	(11.1–12.0)	(12.6–13.4)	(12.8–13.4)				
SBP process (%)	85.2	98.1	96.6	93.0	93.8	98.5	99.3	96.9	<0.001	0.195	0.315	0.327
	(83.6–86.8)	(97.2–99.1)	(95.5–97.6)	(92.2–93.7)	(93.4–94.2)	(98.3–98.6)	(99.2–99.4)	(96.8–97.1)				
SBP mean	151.4	148.7	143.2	143.7	140.4	138.8	137.1	135.8	<0.001	<0.001	<0.001	0.949
(mmHg)	(150.3–152.6)	(147.2–150.3)	(142.1–144.4)	(143.1–144.3)	(140.1–140.7)	(138.6–139.1)	(136.9–137.3)	(135.6–135.9)				
SBP ≥140 mmHg	75.2	69.5	63.1	59.2	53.7	49.4	45.4	41.2	<0.001	<0.001	0.069	0.980
(%)	(73.1–77.4)	(66.2–72.7)	(60.3–65.9)	(57.7–60.7)	(52.8–54.6)	(48.7–50.1)	(44.9–46.0)	(40.7–41.7)				
Hypertension	45.5	53.1	63.3	67.4	70.5	70.7	66.7	66.7	<0.001*	0.578	<0.001	0.632
treatment (%)	(43.2–47.8)	(49.6–56.6)	(60.6–66.1)	(66.1–68.8)	(69.7–71.3)	(70.1–71.4)	(66.1–67.2)	(66.3–67.2)				
Cholesterol-HDL	70.2	96.5	93.6	82.7	86.4	95.9	97.8	95.3	<0.001	0.147	0.432	0.464
ratio process (%)	(68.1–72.3)	(95.2–97.7)	(92.2–95.0)	(81.6–83.8)	(85.9–87.0)	(95.6–96.1)	(97.6–97.9)	(95.1–95.5)				
Cholesterol-HDL	5.1	4.7	4.2	3.9	3.7	3.9	3.8	3.8	<0.001*	0.041	<0.001	0.894
ratio mean	(5.0–5.2)	(4.6–4.7)	(4.1–4.2)	(3.9–3.9)	(3.7–3.7)	(3.9–3.9)	(3.8–3.8)	(3.8–3.8)				
Cholesterol-HDL	75.0	70.1	51.6	42.6	34.8	40.9	38.6	37.5	<0.001*	0.037	0.006	0.930
≥4 (%)	(72.6–77.4)	(66.9–73.4)	(48.7–54.5)	(41.0–44.2)	(33.9–35.7)	(40.2–41.6)	(38.0–39.1)	(37.1–38.0)				
Lipid lowering	17.1	27.6	36.1	41.2	59.4	66.1	70.5	72.1	<0.001	0.129	<0.001	0.931
treatment (%)	(15.4–18.9)	(24.4–30.7)	(33.3–38.8)	(39.8–42.7)	(58.6–60.3)	(65.5–66.8)	(70.0–71.0)	(71.7–72.5)				
ACR	39.6	94.7	86.0	58.4	61.8	83.5	89.5	86.5	<0.001	<0.001	0.018	0.308
Process (%)	(37.4–41.9)	(93.1–96.3)	(84.0–88.0)	(56.9–59.8)	(67.4–68.8)	(83.0–84.0)	(89.1–89.8)	(86.2–86.8)				
Micro-	30.4	27.2	21.7	20.2	16.0	15.5	13.7	14.0	<0.001	0.009	<0.001	0.919
albuminuria (%)	(27.0–33.8)	(24.0–30.4)	(19.1–24.2)	(18.7–21.8)	(15.2–16.8)	(15.0–16.1)	(13.3–14.1)	(13.7–14.4)				
Macro-	8.5	5.7	3.7	3.0	2.4	1.8	1.5	1.2	<0.001	0.019	<0.001	0.947
albuminuria (%)	(6.4–10.5)	(4.1–7.4)	(2.9–5.6)	(2.4–3.7)	(2.0–2.7)	(1.6–2.1)	(1.4–1.7)	(1.1–1.3)				
Foot examined	63.0	99.7	99.9	47.1	74.3	87.8	90.6	85.9	<0.001	0.059	0.561	0.062
(%)	(60.7–65.2)	(99.4–100.0)	(99.8–100.0)	(45.6–48.6)	(73.5–75.1)	(87.3–88.2)	(90.3–91.0)	(85.6–96.3)				
Diminished	19.7	21.5	14.8	13.8	14.0	11.8	9.5	14.2	<0.001*	0.066	0.918	0.570
sensibility (%)	(17.4–22.0)	(18.6–24.4)	(12.8–16.8)	(12.3–15.3)	(13.3–14.7)	(11.4–12.3)	(9.2–9.9)	(13.8–14.5)				
Eye examined (%)	66.2	96.0	93.8	35.4	14.0	60.0	92.4	85.8	<0.001*	<0.001	<0.001	0.280
	(64.0–68.4)	(94.6–97.3)	(92.4–95.2)	(34.0–36.8)	(13.4–14.6)	(59.3–60.7)	(92.1–92.7)	(85.4–86.1)				
DRP (%)	13.1	13.8	11.8	11.4	7.4	4.4	5.7	5.7	<0.001	0.124	0.383	0.818
	(11.2–15.0)	(11.3–16.2)	(9.9–13.7)	(9.8–13.0)	(6.2–8.6)	(4.0–4.8)	(5.4–5.9)	(5.5–6.0)				
BMI	46.7	98.0	96.0	83.3	80.6	94.2	94.8	95.5	<0.001	0.265	<0.001	0.421
Process (%)	(44.4–49.0)	(97.0–99.0)	(94.9–97.1)	(82.2–84.4)	(79.9–81.3)	(93.8–94.5)	(94.5–95.0)	(95.3–95.7)				
BMI mean (kg/m^2^)	29.5	29.8	29.9	30.0	30.0	30.0	30.1	30.2	<0.001	0.6538	<0.001	0.775
	(29.1–29.8)	(29.5–30.2)	(29.6–30.2)	(29.9–30.2)	(29.9–30.1)	(29.9–30.1)	(30.1–30.2)	(30.2–30.3)				
BMI ≤25 kg/m^2^	15.9	13.4	13.2	13.6	14.5	15.1	14.5	14.0	0.028	<0.001	<0.001	0.076
(%)	(13.4–18.4)	(11.0–15.8)	(11.2–15.2)	(12.5–14.7)	(13.8–15.1)	(14.6–15.6)	(14.1–14.9)	(13.6–14.3)				
BMI 25–30 kg/m^2^	43.1	43.2	43.2	41.9	42.1	41.2	40.7	40.4	<0.001	0.055	<0.001	0.898
(%)	(39.8–46.5)	(39.7–46.7)	(40.3–46.1)	(40.3–43.5)	(41.1–43.0)	(40.4–41.9)	(40.1–41.2)	(39.9–40.9)				
BMI >30 kg/m^2^	41.0	43.4	43.6	44.5	43.5	43.7	44.8	45.6	<0.001	0.031	<0.001	0.672
(%)	(37.6–44.3)	(39.9–46.8)	(40.7–46.5)	(42.9–46.1)	(42.5–44.4)	(43.0–44.5)	(44.3–45.4)	(45.2–46.1)				
Smoking process	56.6	99.7	99.7	89.9	89.8	97.7	97.8	96.3	<0.001	<0.001	<0.001	0.408
(%)	(54.3–58.9)	(99.4–100.0)	(99.5–100.0)	(89.0–90.8)	(89.3–90.3)	(97.5–97.9)	(97.7–98.0)	(96.1–96.5)				
smokers (%)	28.1	23.4	23.3	21.9	22.5	21.4	19.3	19.8	<0.001	<0.001	<0.001	0.691
	(25.3–30.9)	(20.5–26.4)	(20.9–25.7)	(20.6–23.2)	(21.8–23.3)	(20.9–22.0)	(18.8–19.7)	(19.4–20.2)				

Data are presented as means, medians or proportions with 95% CIs

^#^ interaction for year and gender

* P value for the quadratic trend was < 0.001 for this variable

Abbreviations: NA: not applicable; DM, diabetes mellitus; OBLD, oral blood glucose-lowering drugs; SBP, systolic blood pressure; HDL, high-density lipoprotein; ACR, albumin-creatinine ratio; DRP, diabetic retinopathy; BMI, body mass index.

### Process measures

A positive trend for men and women <75 years of age was observed for all process measures with a temporary decrease in 2003 and 2006 for most variables (Fig 1 and Table 2). The process measures for foot end eye examination showed another trend with a decrease in the period from 2002 to 2007 and an increase afterwards (Fig 1 and Table 2). Significant trend differences between sexes were observed for the process measures of renal function and eye examination. Significant absolute differences between sexes were observed for the process measures of renal function, eye examination and BMI (Table 2).

### Outcome measures for patients up to 75 years of age

In 1998, 50.9% of the men and 59.5% of the women had an HbA1c >53 mmol/mol. This decreased to 24,9% in women and 26,3% in men in 2008 and increased afterwards to almost 29% in 2013 in both sexes (p for quadratic trend in both sexes <0.001). A small difference in trend between sexes was observed due to the difference in the first years (p for interaction: <0.001). In all years, the use of oral medication was on average 3% higher in men (p for sex <0.001). In the first years, there were more women on insulin compared to men (p for sex < 0.001), whereas in the last years no sex differences was observed (p for interaction <0.001). The number of patients with a systolic blood pressure (SBP) >140 mmHg decreased from 70.1% to 42.0% in men, and from 80.2% to 40.3% in women (p for trend in both sexes <0.001), whereby a small trend difference between sexes was observed due to the difference in the first years (p for interaction <0.001). The use of antihypertensive drugs increased over the years in both sexes in the first half of the study period and decreased slightly afterwards (p for quadratic trend in both sexes <0.001). This increase was slightly higher in men compared to women (p for interaction <0.001) but still these medications were more frequently used in women than men (p for sex <0.001). The percentage of patients with a cholesterol-HDL ratio ≥4 decreased from 76.2% to 39.4% in men and from 73.8% to 29.9% in women in the period from 1998 till 2006 and decreased slightly afterwards in both sexes (p for quadratic trend in both sexes <0.001). A small trend difference between sexes was observed (p for interaction: <0.001) and the target value was in all years more often achieved in women (p for sex <0.001). The use of lipid lowering drugs increased over time and they were more often used by men (p for sex <0.001).

Microalbuminuria was more prevalent in men compared to women (p for sex <0.001). Both micro- and macroalbuminuria decreased over the years in both sexes (p for trend in both sexes <0.001). The prevalence of macroalbuminuria decreased from 10.2% to 1.6% in men and from 7.0% to 0.7% in women (p for interaction = 0.009). Approximately 20% in both sexes had diminished foot sensibility in 1998, which decreased slightly over time to 10.6% in men and 8.3% in women in 2010. In the last years it increased to 15.3% in men and 12.8% in women (p for quadratic trend in both sexes <0.001). Diabetic retinopathy decreased from approximately 13% in 1998 to 6% in 2013 in both sexes (p for trend in both sexes <0.001).

The number of men who had a BMI below 25 kg/m2 decreased slightly over time, whereas for women it remained stable (p for interaction <0.001). In the group of patients with a BMI >25 kg/m2, men were more frequently overweight and women were more frequently obese. Obesity increased from 49.6% to 51.9% in women and from 31.0 to 40.3% in men (p for interaction <0.031). The percentage of smokers decreased from 34.2% to 21.8% in men and from 21.7% to 17.5% in women (p for trend in both sexes <0.001). A trend difference in favor of men was observed (p for interaction <0.001). In the most recent years the prevalence of smokers was 4% higher among men compared to women (p for sex <0.001).

### Outcome measures for patients 75 years and over

In the age category patients of 75 years and over, similar trends were found for HbA1c, BMI, insulin use and for the use of antihypertensive and lipid lowering drugs (S1 and S2 Figs). The target value for systolic blood pressure was more frequently achieved in men compared to women. The sex difference in the cholesterol-HDL ratio was smaller in patients ≥75 years of age compared to the younger patients. The prevalence of micro- and macroalbuminuria was higher in both sexes. The prevalence of diminished sensibility was higher in men compared to women (p for sex <0.001). The prevalence of DRP was higher in women compared to men (p for sex <0.001). In women, a small increase in smoking prevalence was observed during the study period (p for interaction <0.001).

### Post-hoc analyses for the period 2006–2013

In the period 2006–2013 for almost all process and outcome measures the same trends were observed, except for the trends for the outcome measures for HbA1c, renal function, foot examination and eye examination (S3 Table). In this period, the number of men and women with an HbA1c >53 mmol/mol slightly increased. The prevalence of microalbuminuria remained stable in both sexes and the prevalence of diabetic retinopathy and diminished sensibility of the feet increased slightly in both sexes. Significant trend differences between sexes for the outcome measures were only observed for the use of insulin and antihypertensive drugs and for the prevalence of smoking.

### Risk engine

**Fig 4:**
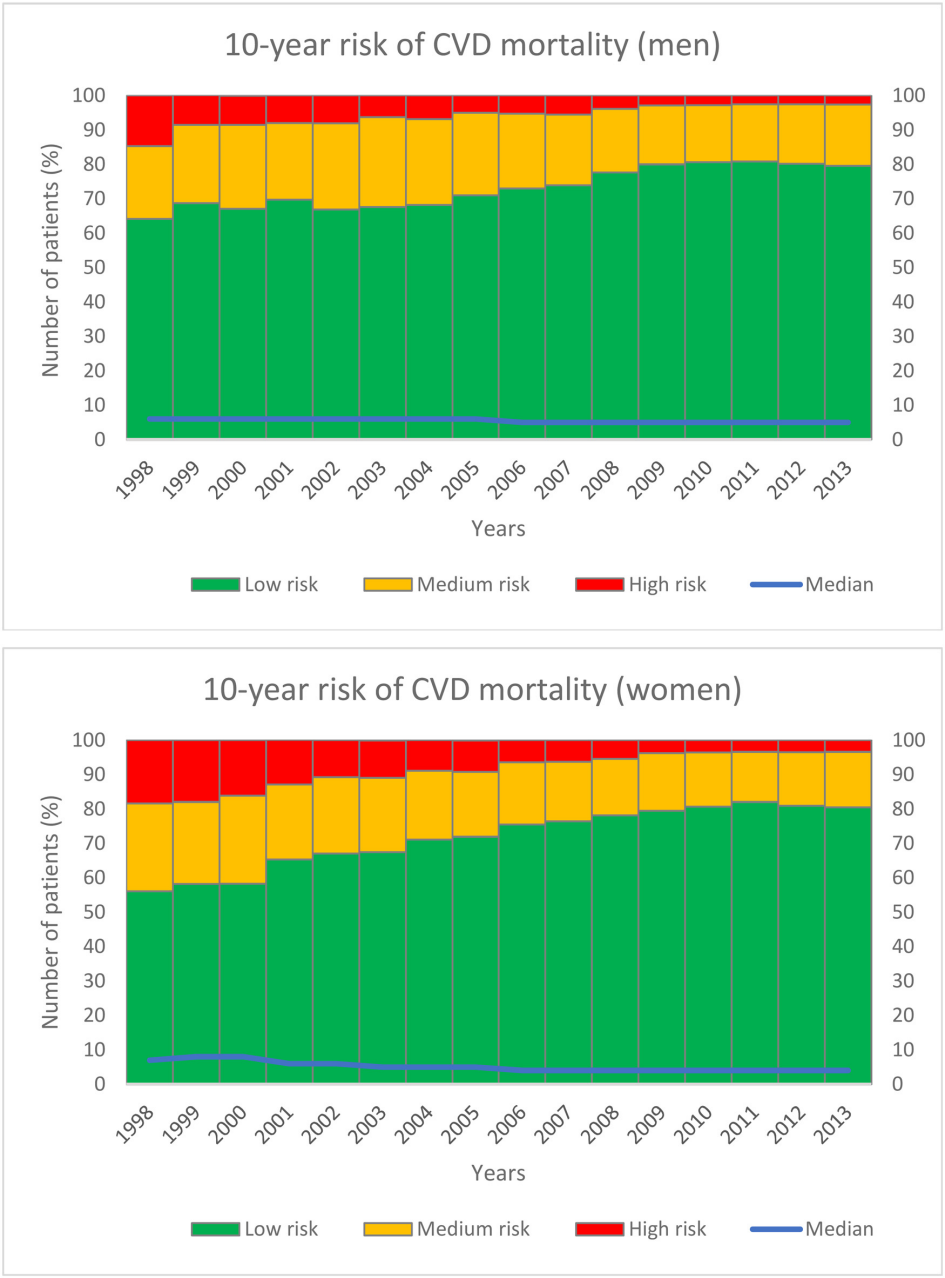
10-years risk of cardiovascular mortality for men and women <75 years of age without a history of CVD.

The estimated 10-year risk of CVD mortality for men and women <75 years of age without a history of CVD diseases is depicted in Fig 4. The number of patients with a low risk for CVD mortality (i.e. <10% 10-years mortality prediction) increased from 64.2% in 1998 to 79.5% in 2013 in men and from 56.1% to 80.5% in women in the same period.

## Discussion

In this study, gender differences in the quality of diabetes care were assessed using process and outcome measures. Possibly relevant trend differences in the improvement of the quality of care between sexes were only observed for the outcome measures of HbA1c, systolic blood pressure, BMI and smoking and these trends converged between sexes over time. No relevant trend differences between sexes were observed for the process measures. All process and outcome parameters improved in the period from 1998 to 2013 in both sexes except for the proportion of patients with normal BMI. At the end of the study period, possibly relevant absolute sex differences were observed for cholesterol-HDL ratio, albuminuria, BMI and smoking. Furthermore, possibly relevant absolute differences in medication use were observed for the use of insulin in the first half of the study period and for antihypertensive drugs and lipid lowering drugs in the whole study period.

In the most recent years almost all process and outcome measures reached a plateau phase, which could imply that for those indicators the (near) maximal attainable goal in relation to effort is already achieved. For the outcome measures of HbA1c, foot examination and eye examination significant deteriorations were observed as shown in the analyses for the period 2006 to 2013. However, these deteriorations are probably not relevant, as the lines in the graphs are almost horizontal.

Many studies have mentioned that cardiovascular risk factors are poorer controlled in women compared to men [3,5,6,14]. In our study, this finding could only be confirmed for BMI in both age groups and for SBP in patients ≥75 years of age. Women had even better cholesterol-HDL ratios, less frequently albuminuria, less frequently diminished sensibility and also smoked less compared to men. The difference between the findings of our study and others could possibly be explained by the well-organized care for diabetes patients in the Netherlands [8]. In this protocol-based care, it is unlikely that women are treated less aggressively. This explanation is widely discussed in a previous study from the same cohort [8]. The explanation could possibly also be found in the interpretation of the results. In previous studies, conclusions concerning sex differences are sometimes based on very small absolute differences and one can doubt about their clinical relevance.

Based on the results of the risk equation, the risk of cardiovascular mortality decreased over time in our study population in both men and women. In 1998, there were more women than men with an intermediate or high mortality risk but the risk at the end of the study period was comparable between sexes. However, this could mean that the risk is relatively higher for women, given the fact that women have a lower mortality risk compared to men in the general population. It remains to be determined in future studies whether the comparable cardiovascular risk will also translate into a comparable mortality risk in our population. In the literature, a higher relative risk for cardiovascular mortality in women with T2D is described [1,2]. Our results indicate that this higher risk in women with T2D is probably not explained by differences in risk factor control between men and women with T2D. However, it might be due to a greater difference in cardiovascular risk factor control between women with and without T2D compared to men with and without T2D [1,15]. Differences in physical activity between men and women could also play an important role [14,16]. Furthermore, women with T2D and coronary atherosclerosis have less obstructive disease compared to men, with higher rates of microvascular coronary dysfunction that may be more difficult to diagnose and treat [17]. Also, a higher prevalence heart failure with preserved ejection fraction (HFpEF) in described in women compared to men with T2D [18]. The higher inflammatory state and unfavorable alterations of the coagulation system in postmenopausal women, may further enhance their higher CVD risk [19]. It remains to be determined whether focussing on these aspects may improve long-term outcomes in women.

Some limitations of this study need to be mentioned. First, the clinical data and the data on medication use in our study are collected by practice nurses and GPs and sent to our Diabetes Centre as part of the yearly benchmark of this project. The quality and reliability of the data are therefore dependent on the accuracy of the data providers. For example, all process measures showed more or less the same trend with a temporary decrease in 2003 and 2006 which could be explained by the introduction of new regions in those specific years. Lack of experience with using the core data set and properly registering of this data in the first year, and as a consequence underreporting on specific data entry positions, may explain the initial lack of data. Second, different laboratories were participating in this project and different methods were used for assessing the blood samples. However, due to the high number of patients in the last years of the project, it is not likely that differences in laboratory methods have influenced the trend results. Third, especially for some process measures, a low model fit was found due to a high variation between years in achieving the process measures. The trend of these process measures should therefore be interpreted with caution. Fourth, whether a difference was described as relevant was based on the interpretation of the authors. Fifth, only the presence of gender differences in process and outcome measures was assessed. Possible explanations for these differences were not investigated. At last, despite of the use of two age groups, still a small difference in mean age between men and women was observed which could theoretically have influenced the results.

In conclusion, quality of care for patients with T2D within this study, has improved considerably in the period 1998–2013 in both sexes. Possibly relevant trend differences in the improvement of the quality of care between sexes were only observed for HbA1c and systolic blood pressure BMI and smoking and all these trends converged over time. The predicted mortality risk improved in men and women and also converged between both sexes over time. Except for BMI in both age groups and systolic blood pressure in patients ≥75 years of age, no evident poorer risk factor control in women compared to men was found.

## Supporting Information

S1 Fig Outcome measures for HbA1c, diabetes treatment, systolic blood pressure, hypertension treatment, cholesterol-HDL ratio, lipid lowering treatment, renal function and foot and eye examination in patients >75 years stratified according to sex. Men: gray line, Women: black striped line.
(TIF)

S2 Fig Outcome measures for BMI and smoking in patients >75 years stratified according to sex. Men: gray line, Women: black striped line.
(TIF)

S1 File SAS output Glimmix outcomes under 75 years of age. (TXT)

S2 File SAS output Glimmix outcomes men and women above 75 years of age. (TXT)

S3 File SAS output Glimmix outcomes under 75 years of age (quadratic analyses). (TXT)

S4 File SAS output Glimmix outcomes above 75 years of age. (TXT)

S5 File SAS output Glimmix outcomes men and women under 75 years of age. (TXT)

S6 File SAS output Glimmix outcomes above 75 years of age (quadratic analyses). (TXT)

S7 File SAS output Glimmix outcomes under 75 years of age 2006–2013. (TXT)

S1 Table Results of the process and outcome measurements for men under 75 years of age. (DOCX)

S2 Table Results of the process and outcome measurements for women under 75 years of age. (DOCX)

S3 Table Results of the process and outcome measurements for the period 2006–2013. (DOCX)
