# Supplementary figures and images for: Sex Differences in the Quality of Diabetes Care in the Netherlands (ZODIAC-45)
Source: PLoS One. 2015 Dec 29;10(12):e0145907. doi: 10.1371/journal.pone.0145907 (PMC4703132; doi:10.1371/journal.pone.0145907)

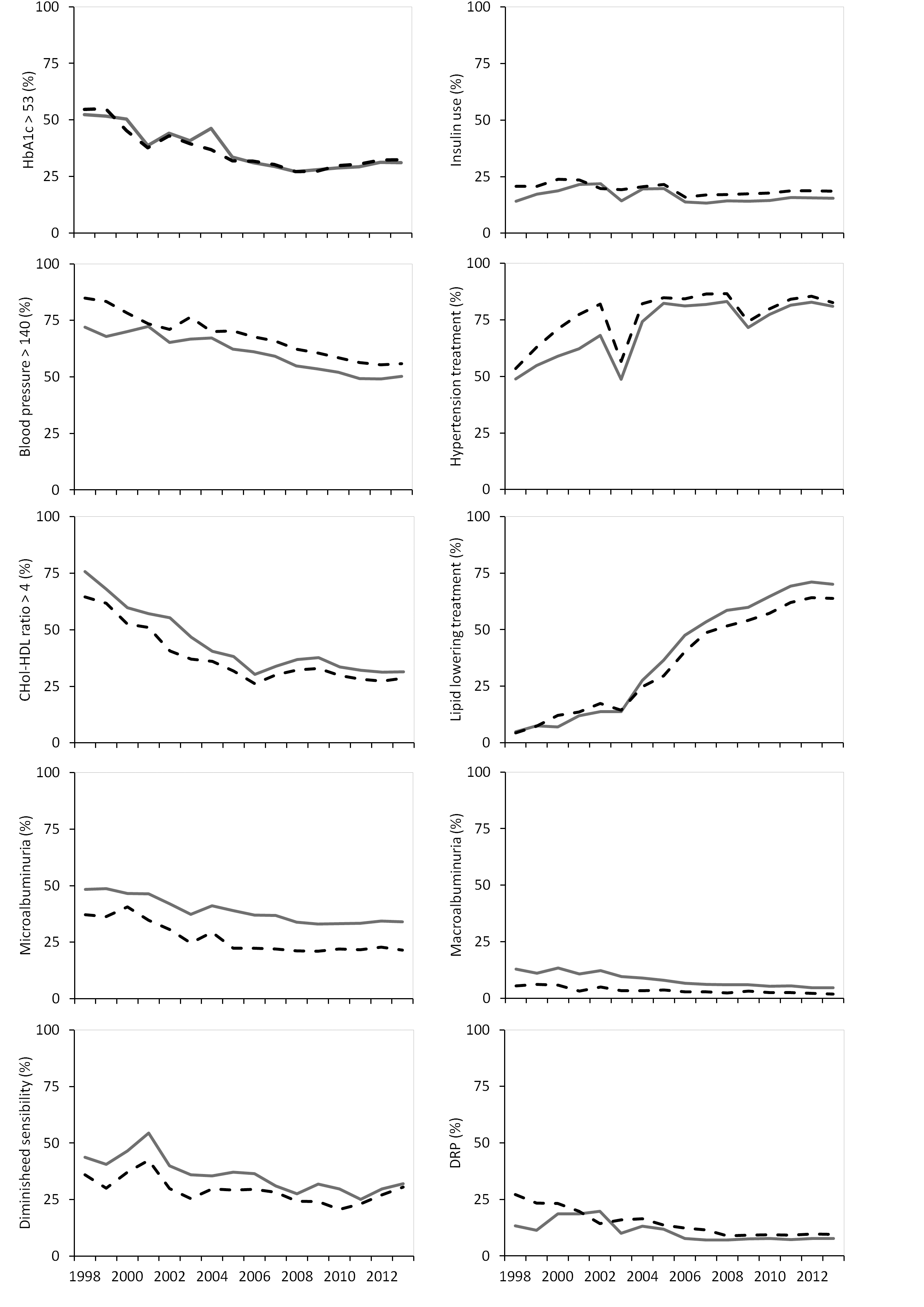

Supplement: S1 Fig — Men: gray line, Women: black striped line. (TIF) [file pone.0145907.s001.tif]

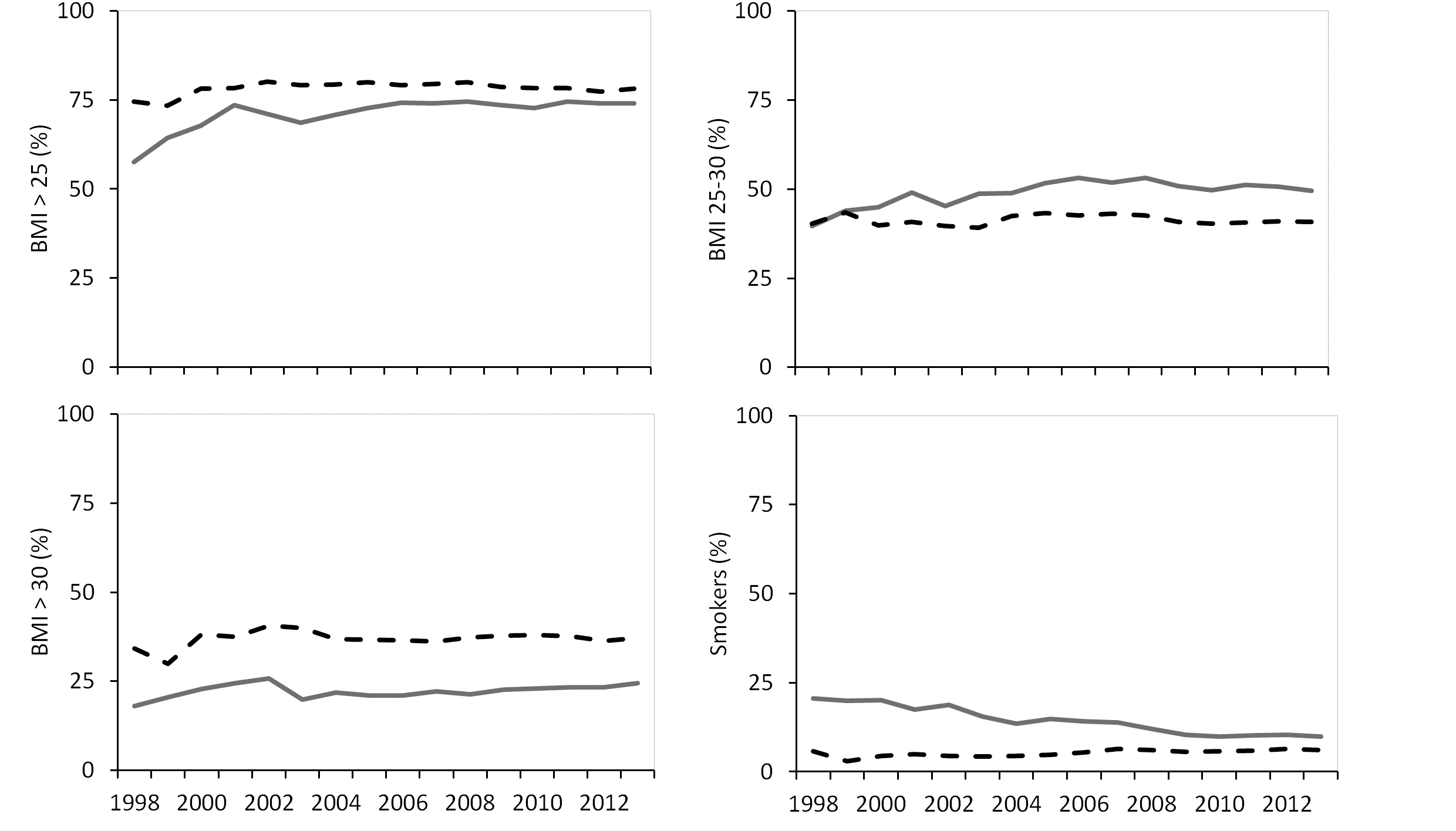

Supplement: S2 Fig — Men: gray line, Women: black striped line. (TIF) [file pone.0145907.s002.tif]
