# Supplementary material for: Sex Differences in the Quality of Diabetes Care in the Netherlands (ZODIAC-45)
Source: PLoS One. 2015 Dec 29;10(12):e0145907. doi: 10.1371/journal.pone.0145907 (PMC4703132; doi:10.1371/journal.pone.0145907)
Supplement: S1 Table — (DOCX) [file pone.0145907.s010.docx]

**S1 Table. Results of the process and outcome measurements for men under 75 years of age.**

| **Variable** | **1998** | **2000** | **2002** | **2004** | **2006** | **2008** | **2010** | **2013** | **P value for trend** |
| --- | --- | --- | --- | --- | --- | --- | --- | --- | --- |
| N | 888 | 391 | 586 | 2286 | 6638 | 10372 | 16146 | 23118 |  |
| Age (years) | 61.0  (60.4 – 61.6) | 61.0  (60.1 – 61.9) | 60.6  (59.8 – 61.3) | 61.2  (60.9 – 61.6) | 61.3  (61.1 – 61.5) | 61.5  (61.4 – 61.7) | 61.5  (61.4 – 61.7) | 62.0  (61.9 – 62.1) | <0.001 |
| DM duration (years) | 4.0  (3.6 – 4.5) | 4.3  (3.8 – 4.9) | 3.6  (3.3 – 4.1) | 4.2  (4.0 – 4.4) | 4.2  (4.1 – 4.3) | 4.8  (4.6 – 4.8) | 5.0  (4.9 – 5.1) | 5.9  (5.8 – 6.0) | <0.001 |
| HbA1c  process (%) | 85.2  (82.9 – 87.6) | 97.7  (96.2 – 99.2) | 91.3  (89.0 – 93.6) | 89.9  (88.7 – 91.1) | 88.2  (87.4 – 89.0) | 95.9  (95.5 – 96.3) | 98.6  (98.5 – 98.8) | 95.4  (95.1 – 95.6) | <0.001 |
| HbA1c mean (mmol/mol) | 56.6  (55.6 – 57.6) | 56.1  (54.7 – 57.6) | 53.2  (52.0 – 54.4) | 51.9  (51.4 – 52.5) | 50.0  (49.7 – 50.3) | 49.4  (49.2 – 49.6) | 50.3  (50.1 – 50.4) | 50.0  (49.8 – 50.1) | <0.001 |
| HbA1c >53 mmol/mol (%) | 50.9  (47.3 – 54.4) | 52.4  (47.3 – 57.4) | 42.1  (37.9 – 46.2) | 35.8  (33.7 – 37.9) | 27.9  (26.7 – 29.0) | 26.3  (25.5 – 27.2) | 28.6  (27.9 – 29.3) | 28.9  (28.3 – 29.5) | <0.001 |
| DM treatment  Diet only (%) | 13.7  (11.5 – 16.0) | 12.3  (9.0 – 15.5) | 18.8  (15.6 – 21.9) | 17.7  (16.2 – 19.3) | 20.2  (19.3 – 21.2) | 19.6  (18.8 – 20.3) | 18.6  (18.0 – 19.2) | 16.2  (15.7 – 16.7) | <0.001 |
| OBLD only (%) | 65.9  (62.8 – 69.0) | 68.3  (63.7 – 72.9) | 63.7  (59.8 – 67.5) | 66.1  (64.1 – 68.0) | 65.4  (64.3 – 66.6) | 65.3  (64.3 – 66.2) | 65.2  (64.5 – 66.0) | 63.9  (63.3 – 64.5) | 0.742 |
| Insulin (%) | 12.0  (9.9 – 14.2) | 12.0  (8.8 – 15.2) | 10.9  (8.4 – 13.4) | 11.5  (10.2 – 12.8) | 10.2  (9.5 – 10.9) | 11.1  (10.5 – 11.7) | 12.4  (11.9 – 13.0) | 12.9  (12.4 – 13.3) | <0.001 |
| SBP process (%) | 83.9  (81.3 – 86.5) | 98.5  (97.2 – 99.7) | 95.7  (94.1 – 97.4) | 93.4  (92.3 – 94.4) | 93.7  (93.1 – 94.3) | 98.4  (98.1 – 98.6) | 99.2  (99.1 – 99.3) | 96.8  (96.6 – 97.1) | <0.001 |
| SBP mean (mmHg) | 148.0  (146.4 – 149.6) | 146.7  (144.5 – 148.8) | 143.5  (141.8 – 145.3) | 143.5  (142.6 – 144.3) | 140.1  (139.7 – 140.5) | 139.0  (138.6 – 139.3) | 137.2  (137.0 – 137.4) | 136.1  (135.9 – 136.3) | <0.001 |
| SBP ≥140 mmHg (%) | 70.1  (66.8 – 73.4) | 66.0  (61.2 – 70.7) | 61.1  (57.1 – 65.2) | 59.1  (57.0 – 61.2) | 53.3  (52.1 – 54.6) | 50.1  (49.1 – 51.0) | 45.7  (44.9 – 46.5) | 42.0  (41.3 – 42.6) | <0.001 |
| Hypertension treatment (%) | 41.8  (38.5 – 45.0) | 50.9  (45.9 – 55.9) | 59.6  (55.6 – 63.5) | 63.9  (61.9 – 65.8) | 68.2  (67.1 – 69.3) | 69.1  (68.2 – 70.0) | 65.7  (65.0 – 66.4) | 65.7  (65.1 – 66.3) | <0.001 |
| Cholesterol-HDL ratio process (%) | 70.6  (67.0 – 74.2) | 95.7  (93.6 – 97.7) | 92.5  (90.3 – 94.7) | 82.8  (81.1 - 84.5) | 85.9  (85.0 – 86.8) | 95.9  (95.5 – 96.3) | 97.9  (96.3 – 96.8) | 95.1  (94.8 – 95.3) | <0.001 |
| Cholesterol-HDL ratio mean | 5.2  (5.0 – 5.3) | 4.8  (4.7 – 4.9) | 4.3  (4.2 – 4.4) | 4.0  (4.0 – 4.1) | 3.8  (3.8 – 3.8) | 4.0  (4.0 – 4.0) | 4.0  (4.0 – 4.0) | 3.9  (3.9 – 4.0) | <0.001 |
| Cholesterol-HDL ≥4 (%) | 76.2  (72.9 – 79.6) | 75.7  (71.3 – 80.0) | 57.9  (53.8 – 62.1) | 48.0  (45.7 – 50.2) | 39.4  (38.2 – 40.7) | 46.0  (45.0 – 47.0) | 43.9  (43.1 – 44.6) | 42.5  (41.9 – 43.2) | <0.001 |
| Lipid lowering treatment (%) | 16.7  (14.2 – 19.1) | 30.7  (26.1 – 35.3) | 40.1  (36.1 – 44.1) | 45.7  (43.6 – 47.7) | 61.7  (60.6 – 62.9) | 67.9  (67.0 – 68.8) | 72.5  (71.8 – 73.1) | 73.6  (73.0 – 74.2) | <0.001 |
| ACR  Process (%) | 36.5  (33.3 – 39.7) | 94.4  (92.1 – 96.7) | 84.6  (81.7 – 87.6) | 58.6  (56.6 – 60.6) | 62.8  (61.6 – 64.0) | 84.2  (83.5 – 84.9) | 90.1  (86.9 – 90.5) | 86.9  (86.5 – 87.4) | <0.001 |
| Micro-  albuminuria (%) | 33.3  (28.2 – 38.5) | 31.7  (27.0 – 36.5) | 28.0  (24.1 – 32.0) | 24.6  (22.3 – 26.9) | 19.1  (17.9 – 20.3) | 19.1  (18.2 – 19.9) | 16.6  (16.0 – 17.2) | 16.8  (16.3 – 17.3) | <0.001 |
| Macro-  albuminuria (%) | 10.2  (6.9 – 13.5) | 7.6  (4.9 – 10.3) | 4.8  (3.0 – 6.7) | 3.5  (2.5 – 4.5) | 3.4  (2.9 – 4.0) | 2.4  (2.1 – 2.8) | 2.1  (1.9 – 2.4) | 1.6  (1.5 – 1.8) | <0.001 |
| Foot examined (%) | 62.3  (59.1 – 65.5) | 99.5  (98.8 – 100.0) | 100.0  (100.0 – 100.0) | 47.4  (45.4 – 49.5) | 74.4  (73.3 – 75.4) | 87.0  (86.4 – 87.7) | 90.3  (89.8 – 90.7) | 85.5  (85.0 – 85.9) | <0.001 |
| Diminished sensibility (%) | 19.5  (16.2 – 22.8) | 21.6  (17.5 – 25.6) | 15.6  (12.6 – 18.5) | 14.6  (12.5 – 16.7) | 15.5  (14.5 – 16.5) | 13.0  (12.3 – 13.7) | 10.6  (10.1 – 11.1) | 15.3  (14.8 – 15.8) | <0.001 |
| Eye examined (%) | 66.1  (63.0 – 69.2) | 95.7  (93.6 – 97.7) | 93.9  (91.9 – 95.8) | 35.5  (33.5 -37.4) | 14.4  (13.5 – 15.2) | 60.2  (59.2 – 61.1) | 92.0  (91.6 – 92.4) | 85.6  (85.2 – 86.1) | <0.001 |
| DRP (%) | 13.2  (9.7 – 16.7) | 12.1  (8.1 – 16.1) | 10.2  (7.0 – 13.4) | 10.6  (8.2 – 13.0) | 7.4  (5.7 – 9.2) | 5.0  (4.5 – 5.6) | 6.2  (5.8 – 6.6) | 6.1  (5.8 – 6.4) | <0.001 |
| BMI  Process (%) | 43.6  (40.3 – 46.8) | 98.2  (96.9 – 99.5) | 94.9  (93.1 – 96.7) | 84.0  (82.5 – 85.5) | 80.8  (79.8 – 81.7) | 94.1  (93.7 – 94.6) | 95.0  (94.7 – 95.4) | 95.3  (95.0 – 95.6) | <0.001 |
| BMI mean (kg/m^2^) | 28.5  (28.1 – 29.0) | 29.2  (28.7 – 29.6) | 28.9  (28.6 – 29.3) | 29.1  (28.9 – 29.3) | 29.3  (29.2 – 29.4) | 29.3  (29.2 – 29.4) | 29.5  (29.4 – 29.5) | 29.6  (29.5 – 29.6) | <0.001 |
| BMI <25 kg/m^2^ (%) | 18.6  (14.7 – 22.5) | 13.3  (9.9 – 16.7) | 14.9  (12.0 – 17.9) | 14.7  (13.1 – 16.3) | 14.8  (13.9 – 15.8) | 15.3  (14.6 – 16.1) | 14.3  (13.7 – 14.8) | 13.9  (13.4 – 14.3) | 0.021 |
| BMI 25-30 kg/m^2^ (%) | 50.4  (45.4 – 55.4) | 49.2  (44.2 – 54.2) | 50.2  (46.0 – 54.3) | 49.0  (46.7 – 51.2) | 48.1  (46.8 – 49.5) | 47.0  (46.0 – 48.0) | 46.7  (45.9 – 47.5) | 45.8  (45.2 – 46.5) | <0.001 |
| BMI >30 kg/m^2^ (%) | 31.0  (26.4 – 35.6) | 37.5  (32.7 – 42.3) | 34.9  (30.9 – 38.9) | 36.3  (34.1 – 38.4) | 37.0  (35.7 – 38.3) | 37.7  (36.7 – 38.6) | 39.0  (38.3 – 39.8) | 40.3  (39.7 – 40.9) | <0.001 |
| Smoking process (%) | 58.2  (55.0 – 61.5) | 100.0  (100.0 – 100.0) | 99.7  (99.2 – 100.0) | 90.0  (88.8 – 91.3) | 89.9  (89.2 – 90.7) | 97.6  (97.3 – 97.9) | 97.8  (97.6 – 98.0) | 96.1  (95.8 – 96.3) | <0.001 |
| Smokers (%) | 34.2  (30.1 – 38.3) | 29.7  (25.1 – 34.2) | 27.4  (23.8 – 31.0) | 26.1  (24.2 – 28.0) | 26.2  (25.1 – 27.3) | 23.9  (23.1 – 24.8) | 21.3  (20.7 – 21.9) | 21.8  (21.2 – 22.3) | <0.001 |

Data are presented as means, medians or proportions with 95% CIs.

Abbreviations: DM, diabetes mellitus; OBLD, oral blood glucose-lowering drugs; SBP, systolic blood pressure; HDL, high-density lipoprotein; ACR, albumin-creatinine ratio; DRP, diabetic retinopathy; BMI, body mass index.
