# Supplementary material for: Sex Differences in the Quality of Diabetes Care in the Netherlands (ZODIAC-45)
Source: PLoS One. 2015 Dec 29;10(12):e0145907. doi: 10.1371/journal.pone.0145907 (PMC4703132; doi:10.1371/journal.pone.0145907)
Supplement: S2 Table — (DOCX) [file pone.0145907.s011.docx]

**S2 Table. Results of the process and outcome measurements for women under 75 years of age.**

| **Variable** | **1998** | **2000** | **2002** | **2004** | **2006** | **2008** | **2010** | **2013** | **P value for trend** |
| --- | --- | --- | --- | --- | --- | --- | --- | --- | --- |
| N | 903 | 400 | 606 | 2185 | 6195 | 9500 | 14192 | 19523 |  |
| Age (years) | 63.3  (62.7 – 63.8) | 63.2  (62.4 – 64.0) | 62.6  (61.9 – 63.3) | 62.5  (62.2 – 62.9) | 62.2  (61.9 – 62.4) | 62.2  (62.0 – 62.4) | 62.2  (62.0 – 62.3) | 62.4  (62.3 – 62.5) | <0.001 |
| DM duration (years) | 4.8  (4.5 – 5.1) | 5.3  (4.7 – 6.0) | 4.2  (3.9 – 4.7) | 4.5  (4.3 – 4.7) | 4.4  (4.2 – 4.5) | 4.9  (4.8 – 5.0) | 5.2  (5.2 – 5.3) | 6.2  (6.1 – 6.3) | <0.001 |
| HbA1c  process (%) | 88.4  (86.3 – 90.5) | 98.5  (97.3 – 99.7) | 94.6  (92.7 – 96.4) | 90.0  (88.8 – 91.3) | 89.4  (88.6 – 90.1) | 96.2  (95.8 – 96.6) | 98.6  (98.4 – 98.8) | 95.8  (95.5 – 96.1) | <0.001 |
| HbA1c mean (mmol/mol) | 58.4  (57.4 – 59.3) | 57.5  (56.2 – 58.8) | 54.4  (53.4 – 55.5) | 52.2  (51.7 – 52.7) | 49.8  (49.6 – 50.1) | 48.9  (48.7 – 49.1) | 50.0  (49.9 – 50.2) | 50.1  (50.0 – 50.3) | <0.001 |
| HbA1c >53 mmol/mol (%) | 59.5  (56.1 – 62.9) | 57.4  (52.5 – 62.2) | 46.9  (42.9 – 51.0) | 37.3  (35.1 – 39.4) | 27.0  (25.8 – 28.1) | 24.9  (24.0 – 25.8) | 27.0  (26.3 – 27.8) | 28.3  (27.7 – 29.0) | <0.001 |
| DM treatment  Diet only (%) | 13.8  (11.6 – 16.1) | 13.8  (10.4 – 17.1) | 20.5  (17.3 – 23.7) | 19.0  (17.3 – 20.6) | 22.5  (21.5 – 23.6) | 21.5  (20.6 – 22.3) | 20.7  (20.0 – 21.3) | 18.5  (17.9 – 19.0) | <0.001 |
| OBLD only (%) | 66.1  (63.0 – 69.2) | 66.0  (61.4 – 70.6) | 59.4  (55.5 – 63.3) | 60.4  (58.4 – 62.5) | 62.4  (62.1 – 63.6) | 62.7  (61.7 – 63.6) | 62.5  (61.7 – 63.3) | 61.5  (60.8 – 62.2) | 0.838 |
| Insulin (%) | 15.0  (12.6 – 17.3) | 17.0  (13.3 – 20.7) | 17.2  (14.2 – 20.2) | 17.5  (15.9 – 19.1) | 11.8  (11.0 – 12.6) | 12.1  (11.4 – 12.7) | 13.6  (13.1 – 14.2) | 13.3  (12.9 – 13.8) | 0.513 |
| SBP process (%) | 86.5  (84.1 – 88.9) | 97.8  (96.3 – 99.2) | 97.4  (96.1 – 98.7) | 92.5  (91.4 – 93.7) | 93.9  (93.3 – 94.5) | 98.5  (98.3 – 98.8) | 99.4  (99.2 – 99.5) | 97.0  (96.8 – 97.3) | <0.001 |
| SBP mean (mmHg) | 154.7  (153.1 – 156.3) | 150.7  (148.5 – 153.0) | 142.9  (141.4 – 144.5) | 143.9  (143.1 – 144.8) | 140.7  (140.3 – 141.2) | 138.7  (138.4 – 139.1) | 137.0  (136.7 – 137.2) | 135.4  (135.1 – 135.6) | <0.001 |
| SBP ≥140 mmHg (%) | 80.2  (77.4 – 83.0) | 72.9  (68.5 – 77.3) | 64.9  (61.1 – 68.8) | 59.2  (57.1 – 61.4) | 54.1  (52.8 – 55.4) | 48.8  (47.7 – 49.8) | 45.1  (44.3 – 46.0) | 40.3  (39.6 – 41.0) | <0.001 |
| Hypertension treatment (%) | 49.2  (45.9 – 52.4) | 55.3  (50.4 – 60.1) | 67.0  (63.3 – 70.7) | 71.2  (69.3 – 73.1) | 73.0  (71.9 – 74.1) | 72.5  (71.6 – 73.4) | 67.8  (67.0 – 68.6) | 67.9  (67.3 – 68.6) | <0.001 |
| Cholesterol-HDL ratio process (%) | 69.8  (66.2 – 73.4) | 97.3  (95.6 – 98.9) | 94.7  (92.9 – 96.5) | 82.5  (80.8 – 84.3) | 87.0  (86.1 – 87.9) | 95.9  (95.5 – 96.3) | 97.6  (96.5 – 97.0) | 95.6  (95.3 – 95.9) | <0.001 |
| Cholesterol-HDL ratio mean | 5.1  (4.9 – 5.2) | 4.5  (4.4 – 4.7) | 4.0  (3.9 – 4.1) | 3.8  (3.7 – 3.8) | 3.6  (3.5 – 3.6) | 3.7  (3.7 – 3.8) | 3.7  (3.6 – 3.7) | 3.6  (3.6 – 3.6) | <0.001 |
| Cholesterol-HDL ≥4 (%) | 73.8  (70.4 – 77.2) | 64.8  (60.0 – 69.5) | 45.6  (41.6 -49.7) | 37.0  (34.8 – 39.2) | 29.9  (28.6 – 31.1) | 35.3  (34.4 – 36.3) | 32.6  (31.8 – 33.3) | 31.7  (31.0 – 32.4) | <0.001 |
| Lipid lowering treatment (%) | 17.6  (15.1 – 20.1) | 24.5  (20.3 – 28.7) | 32.2  (28.5 – 35.9) | 36.6  (34.6 – 38.6) | 57.0  (55.7 – 58.2) | 64.2  (63.2 – 65.2) | 68.2  (67.5 – 69.0) | 70.4  (69.7 – 71.0) | <0.001 |
| ACR  Process (%) | 42.7  (39.5 – 46.0) | 95.0  (92.9 – 97.1) | 87.3  (84.6 – 89.9) | 58.1  (56.1 – 60.2) | 60.7  (59.5 – 62.0) | 82.8  (82.0 – 83.6) | 88.8  (88.3 – 89.3) | 86.0  (85.5 – 86.5) | <0.001 |
| Micro-  albuminuria (%) | 28.0  (23.5 – 32.5) | 22.9  (18.7 – 27.1) | 15.7  (12.6 – 18.8) | 15.7  (13.7 – 17.7) | 12.6  (11.6 – 13.7) | 11.6  (10.9 – 12.3) | 10.3  (9.8 – 10.9) | 10.7  (10.2 – 11.1) | <0.001 |
| Macro-  albuminuria (%) | 7.0  (4.5 – 9.5) | 3.9  (2.0 – 5.9) | 2.6  (1.3 – 4.0) | 2.5  (1.7 – 3.4) | 1.2  (0.9 – 1.6) | 1.2  (0.9 – 1.4) | 0.9  (0.7 – 1.0) | 0.7  (0.5 – 0.8) | <0.001 |
| Foot examined (%) | 63.7  (60.5 – 66.8) | 100.0  (100.0 – 100.0) | 99.8  (99.5 – 100.0) | 46.8  (44.7 – 48.9) | 74.2  (73.1 – 75.3) | 88.6  (87.9 – 89.2) | 91.0  (90.6 – 91.5) | 86.5  (86.0 – 87.0) | <0.001 |
| Diminished sensibility (%) | 19.9  (16.3 – 23.5) | 21.4  (17.4 – 25.5) | 14.0  (112 – 16.8) | 12.9  (10.8 – 15.1) | 12.2  (10.3 – 14.2) | 10.5  (9.3 – 11.7) | 8.3  (6.7 – 9.9) | 12.8  (12.3 – 13.4) | <0.001 |
| Eye examined (%) | 66.2  (63.1 – 69.3) | 96.3  (94.4 – 98.1) | 93.7  (91.8 – 95.7) | 35.3  (33.3 – 37.3) | 13.5  (12.7 – 14.4) | 59.8  (58.9 – 60.8) | 92.8  (92.4 – 93.2) | 86.0  (85.5 – 86.5) | <0.001 |
| DRP (%) | 13.1  (9.5 – 16.7) | 15.5  (11.0 – 20.0) | 13.2  (9.7 – 16.8) | 12.3  (9.6 – 15.0) | 7.4  (5.5 – 9.3) | 3.7  (3.2 – 4.3) | 5.1  (4.7 – 5.5) | 5.3  (4.9 – 5.6) | <0.001 |
| BMI  Process (%) | 49.8  (46.6 – 53.1) | 97.8  (96.3 – 99.2) | 97.0  (95.7 – 98.4) | 82.6  (81.0 – 84.2) | 80.4  (79.4 – 81.4) | 94.2  (93.7 – 94.7) | 94.5  (94.2 – 94.9) | 95.7  (95.4 – 96.0) | <0.001 |
| BMI mean (kg/m^2^) | 30.3  (29.8 – 30.7) | 30.5  (30.0 – 31.0) | 30.9  (30.4 – 31.3) | 31.0  (30.7 – 31.3) | 30.8  (30.6 – 31.0) | 30.8  (30.7 – 30.9) | 30.9  (30.8 – 31.0) | 31.0  (30.9 – 31.1) | <0.001 |
| BMI <25 kg/m^2^ (%) | 13.6  (10.4 – 16.7) | 13.6  (10.2 – 16.9) | 11.6  (9.0 – 14.1) | 12.5  (10.9 – 14.0) | 14.1  (13.1 – 15.0) | 14.8  (14.1 – 15.6) | 14.7  (14.1 – 15.3) | 14.1  (13.6 – 14.6) | 0.341 |
| BMI 25-30 kg/m^2^ (%) | 36.9  (32.4 – 41.3) | 37.3  (32.5 – 42.1) | 36.6  (32.7 – 40.5) | 34.3  (32.2 – 36.5) | 35.5  (34.2 – 36.9) | 34.8  (33.8 – 35.8) | 33.8  (33.0 – 34.6) | 33.9  (33.3 – 34.6) | <0.001 |
| BMI >30 kg/m^2^ (%) | 49.6  (44.9 – 54.2) | 49.1  (44.1 – 54.1) | 51.9  (47.8 – 55.9) | 53.2  (50.9 – 55.5) | 50.4  (49.0 – 51.8) | 50.4  (49.3 – 51.4) | 51.5  (50.6 – 52.3) | 51.9  (51.2 – 52.7) | <0.001 |
| Smoking process (%) | 55.0  (50.7 – 59.4) | 99.5  (98.8 – 100.0) | 99.8  (99.5 – 100.0) | 89.7  (88.4 – 91.0) | 89.7  (88.9 – 90.5) | 97.7  (97.4 – 98.0) | 97.9  (97.7 – 98.1) | 96.6  (96.3 – 96.8) | <0.001 |
| Smokers (%) | 21.7  (18.1 – 25.4) | 17.3  (13.6 – 21.1) | 19.3  (16.2 – 22.5) | 17.5  (15.8 – 19.2) | 18.6  (17.6 – 19.6) | 18.7  (17.9 – 19.5) | 17.0  (16.4 – 17.6) | 17.5  (17.0 – 18.1) | <0.001 |

Data are presented as means, medians or proportions with 95% CIs.

Abbreviations: DM, diabetes mellitus; OBLD, oral blood glucose-lowering drugs; SBP, systolic blood pressure; HDL, high-density lipoprotein; ACR, albumin-creatinine ratio; DRP, diabetic retinopathy; BMI, body mass index.
