# Supplementary material for: Sex Differences in the Quality of Diabetes Care in the Netherlands (ZODIAC-45)
Source: PLoS One. 2015 Dec 29;10(12):e0145907. doi: 10.1371/journal.pone.0145907 (PMC4703132; doi:10.1371/journal.pone.0145907)
Supplement: S3 Table — (DOCX) [file pone.0145907.s012.docx]

**S4 Table. Results of the process and outcome measurements for patients under 75 years of age for the period 2006-2013.**

| **Variable** | **2006** | **2008** | **2010** | **2013** | **P value for linear trend** | **P value for interaction^#^** | **P value for gender** |
| --- | --- | --- | --- | --- | --- | --- | --- |
| N | 12833 | 19872 | 30338 | 42641 | NA | NA | NA |
| Age (years) | 61.7  (61.6 – 61.9) | 61.9  (61.7 – 62.0) | 61.8  (61.7 – 61.9) | 62.2  (62.1 – 62.3) | <0.001 | <0.001 | <0.001 |
| Sex  (% female) | 48.3  (47.4 – 49.1) | 47.8  (47.1 – 48.5) | 46.8  (46.2 – 47.3) | 45.8  (45.3 – 46.3) | <0.001 | NA | <0.001 |
| DM duration (years) | 4.3  (4.2 – 4.4) | 4.8  (4.8 – 4.9) | 5.1  (5.0 – 5.2) | 6.0  (6.0 – 6.1) | <0.001 | 0.020 | 0.171 |
| HbA1c  process (%) | 88.7  (88.2 – 89.3) | 96.0  (95.7 – 96.3) | 98.6  (98.5 – 98.8) | 95.6  (95.4 – 95.8) | <0.001 | 0.398 | 0.861 |
| HbA1c mean (mmol/mol) | 49.9  (49.7 – 50.1) | 49.2  (49.0 – 49.3) | 50.2  (50.0 – 50.2) | 50.2  (50.1 – 50.3) | <0.001 | 0.557 | 0.151 |
| HbA1c >53 mmol/mol (%) | 27.4  (26.6 – 28.3) | 25.7  (25.0 – 26.3) | 27.9  (27.4 – 28.4) | 28.6  (28.2 – 29.1) | <0.001 | 0.175 | 0.027 |
| DM treatment  Diet only (%) | 21.4  (20.6 – 22.1) | 20.5  (19.9 – 21.0) | 19.6  (19.1 – 20.0) | 17.2  (16.9 – 17.6) | <0.001 | 0.462 | <0.001 |
| OBLD only (%) | 63.9  (63.1 – 64.3) | 64.0  (63.3 – 64.7) | 64.0  (63.4 – 64.5) | 62.8  (62.3 – 63.2) | <0.001 | 0.394 | <0.001 |
| Insulin (%) | 11.0  (10.4 – 11.5) | 11.5  (11.1 – 12.0) | 13.0  (12.6 – 13.4) | 13.1  (12.8 – 13.4) | <0.001 | <0.001 | <0.001 |
| SBP process (%) | 93.8  (93.4 – 94.2) | 98.5  (98.3 – 98.6) | 99.3  (99.2 – 99.4) | 96.9  (96.8 – 97.1) | <0.001 | 0.203 | 0.280 |
| SBP mean (mmHg) | 140.4  (140.1 – 140.7) | 138.8  (138.6 – 139.1) | 137.1  (136.9 – 137.3) | 135.8  (135.6 – 135.9) | <0.001 | 0.082 | 0.853 |
| SBP ≥140 mmHg (%) | 53.7  (52.8 – 54.6) | 49.4  (48.7 – 50.1) | 45.4  (44.9 – 46.0) | 41.2  (40.7 – 41.7) | <0.001 | 0.833 | 0.112 |
| Hypertension treatment (%) | 70.5  (69.7 – 71.3) | 70.7  (70.1 – 71.4) | 66.7  (66.1 – 67.2) | 66.7  (66.3 – 67.2) | <0.001 | <0.001 | <0.001 |
| Cholesterol-HDL ratio process (%) | 86.4  (85.9 – 87.0) | 95.9  (95.6 – 96.1) | 97.8  (97.6 – 97.9) | 95.3  (95.1 – 95.5) | <0.001 | 0.016 | 0.042 |
| Cholesterol-HDL ratio mean | 3.7  (3.7 – 3.7) | 3.9  (3.9 – 3.9) | 3.8  (3.8 – 3.8) | 3.8  (3.8 – 3.8) | <0.001 | 0.392 | <0.001 |
| Cholesterol-HDL ≥4 (%) | 34.8  (33.9 – 35.7) | 40.9  (40.2 – 41.6) | 38.6  (38.0 – 39.1) | 37.5  (37.1 – 38.0) | <0.001 | 0.392 | <0.001 |
| Lipid lowering treatment (%) | 59.4  (58.6 – 60.3) | 66.1  (65.5 – 66.8) | 70.5  (70.0 – 71.0) | 72.1  (71.7 – 72.5) | <0.001 | 0.648 | <0.001 |
| ACR  Process (%) | 61.8  (67.4 – 68.8) | 83.5  (83.0 – 84.0) | 89.5  (89.1 – 89.8) | 86.5  (86.2 – 86.8) | <0.001 | 0.175 | <0.001 |
| Micro-  albuminuria (%) | 16.0  (15.2 – 16.8) | 15.5  (15.0 – 16.1) | 13.7  (13.3 – 14.1) | 14.0  (13.7 – 14.4) | 0.706 | 0.248 | <0.001 |
| Macro-  albuminuria (%) | 2.4  (2.0 – 2.7) | 1.8  (1.6 – 2.1) | 1.5  (1.4 – 1.7) | 1.2  (1.1 – 1.3) | <0.001 | 0.337 | <0.001 |
| Foot examined (%) | 74.3  (73.5 – 75.1) | 87.8  (87.3 – 88.2) | 90.6  (90.3 – 91.0) | 85.9  (85.6 – 96.3) | <0.001 | 0.236 | 0.559 |
| Diminished sensibility (%) | 14.0  (13.3 – 14.7) | 11.8  (11.4 – 12.3) | 9.5  (9.2 – 9.9) | 14.2  (13.8 – 14.5) | <0.001 | 0.475 | 0.019 |
| Eye examined (%) | 14.0  (13.4 – 14.6) | 60.0  (59.3 – 60.7) | 92.4  (92.1 – 92.7) | 85.8  (85.4 – 86.1) | <0.001 | <0.001 | <0.001 |
| DRP (%) | 7.4  (6.2 – 8.6) | 4.4  (4.0 – 4.8) | 5.7  (5.4 – 5.9) | 5.7  (5.5 – 6.0) | <0.001 | 0.492 | 0.108 |
| BMI  Process (%) | 80.6  (79.9 – 81.3) | 94.2  (93.8 – 94.5) | 94.8  (94.5 – 95.0) | 95.5  (95.3 – 95.7) | <0.001 | 0.333 | 0.427 |
| BMI mean (kg/m^2^) | 30.0  (29.9 – 30.1) | 30.0  (29.9 – 30.1) | 30.1  (30.1 – 30.2) | 30.2  (30.2 – 30.3) | <0.001 | 0.289 | <0.001 |
| BMI ≤25 kg/m^2^ (%) | 14.5  (13.8 – 15.1) | 15.1  (14.6 – 15.6) | 14.5  (14.1 – 14.9) | 14.0  (13.6 – 14.3) | 0.023 | 0.556 | 0.380 |
| BMI 25-30 kg/m^2^ (%) | 42.1  (41.1 – 43.0) | 41.2  (40.4 – 41.9) | 40.7  (40.1 – 41.2) | 40.4  (39.9 – 40.9) | <0.001 | 0.993 | <0.001 |
| BMI >30 kg/m^2^ (%) | 43.5  (42.5 – 44.4) | 43.7  (43.0 – 44.5) | 44.8  (44.3 – 45.4) | 45.6  (45.2 – 46.1) | <0.001 | 0.580 | <0.001 |
| Smoking process (%) | 89.8  (89.3 – 90.3) | 97.7  (97.5 – 97.9) | 97.8  (97.7 – 98.0) | 96.3  (96.1 – 96.5) | <0.001 | 0.092 | 0.2301 |
| smokers (%) | 22.5  (21.8 – 23.3) | 21.4  (20.9 – 22.0) | 19.3  (18.8 – 19.7) | 19.8  (19.4 – 20.2) | <0.001 | 0.006 | <0.001 |

Data are presented as means, medians or proportions with 95% CIs. ^#^ interaction for year and gender

Abbreviations: NA: not applicable; DM, diabetes mellitus; OBLD, oral blood glucose-lowering drugs; SBP, systolic blood pressure; HDL, high-density lipoprotein; ACR, albumin-creatinine ratio; DRP, diabetic retinopathy; BMI, body mass index.
